# Midwifery students’ satisfaction with simulation-based education and associated factors among governmental universities in Amhara region, Ethiopia 2022

**DOI:** 10.1186/s12909-024-05974-2

**Published:** 2024-09-30

**Authors:** Fiker Chane Gebrekidan, Amlaku Mulat Aweke, Getahun Belay Gella, Yibeltal Alemu Bekele

**Affiliations:** 1 School of Midwifery, College of Health Sciences, Woldia University, Woldia, Ethiopia; 2 Department of Midwifery, College of Medicine and Health Sciences, Bahir Dar University, Bahir Dar, Ethiopia; 3 Department of Reproductive Health and Population Studies, College of Medicine and Health Sciences, Bahir Dar University, Bahir Dar, Ethiopia

**Keywords:** Midwifery, Ethiopia, Student satisfaction, Simulation-based education

## Abstract

**Background:**

Simulation is a method of learning in which the learner experiences a simulated situation instead of being physically present in the clinical area. Exposing students to simulation-based education improves learners’ clinical competence and ability to make decisions, which are crucial for today’s health workforce. When given the proper circumstances, such as receiving feedback on their performance, having the chance for repeated practice, and having simulation as a core component of the curriculum, simulated instruction greatly aids in learning. Although previous studies have been conducted in this area, there are gaps in determining the factors related to their learning environment and design characteristics.

**Method:**

An institution-based cross-sectional study was conducted on 413 midwifery students in Amhara region universities from August 1–30, 2022. Study participants were selected via a simple random sampling technique. Data were collected from third and fourth-year undergraduate midwifery students through a self-administered questionnaire. Epi Data version 4.6 and Statistical Package for Social Sciences version 26 were used for data entry and analysis, respectively. Bivariable and multivariable logistic regression analyses were employed; a P-value of less than 0.05 was considered statistically significant in the study.

**Result:**

This study revealed that 84.7% (95% CI: 81.1–88.3) of midwifery students in Amhara region universities were satisfied with simulation-based education. Year of study [AOR: 2.936; 95% CI (1.531–5.631)], adequate support [AOR: 2.903; 95% CI (1.217–6.922)], availability of instructors [AOR = 2.861, 95% CI (1.078–7.591)], and provision of checklists [AOR: 2.326; 95% CI (1.143–4.734)] were found to be statistically significant variables.

**Conclusion:**

This study revealed undergraduate midwifery students were more satisfied with simulation-based education compared with previous studies conducted in Ethiopia. Predictor variables such as year of study, support, instructor availability, and provision of checklists were significantly associated with student satisfaction. Hence, midwifery departments should strengthen the support given by staff, encourage their instructors to be available during simulations, improve the utilization and provision of checklists to students as much as possible.

**Supplementary Information:**

The online version contains supplementary material available at 10.1186/s12909-024-05974-2.

## Introduction

The replacement of real experiences with guided experiences that evoke the real world in a fully participatory way is called simulation [1]. Simulation enables the learner to experience a situation without being physically present clinically [2]. Any activity that uses simulative aides (such as simulated person, object, or set of circumstances) to imitate clinical circumstances for educational purposes is referred to as simulation-based education (SBE) or practice [3]. In the fields of medicine and health sciences, simulation is a common educational method, as it is critical for blending theoretical and clinical knowledge [4–8]. It is thought that the second half of the 20th century marked the beginning of the contemporary era of simulation. The field of simulation use has undergone exponential expansion in the last twenty years in terms of both development and application [9, 10]. However, some argue that it has been used for centuries, yet one in ten patients is still found to be harmed during hospitalization, even in high-income nations [11]. Globally, the use of simulation in higher education curricula has increased significantly. This growth is fueled by a variety of factors, which raises crucial considerations about how we see simulation pedagogies [3].

Simulation-based education is becoming a preferred teaching method with a wide range of benefits such as promoting students’ knowledge, skill proficiency, critical thinking, and satisfaction. Health workers who receive team training that includes simulation have been linked to better results and fewer adverse effects [11]. Therefore, it is crucial for patients’ and childbearing women’s safety both now and in the future. Patient safety during the delivery of safe and high-quality health services is a prerequisite for strengthening healthcare systems and progressing toward effective universal health coverage (UHC) under Sustainable Development Goal 3 [12].

Exposing students to SBE interventions has a positive effect on students’ learning. Learners can build the knowledge, skills, and abilities needed to make informed decisions and solve problems. SBE improves learners’ clinical competence, self-confidence, and satisfaction, which are crucial for the health workforce [13–15]. It has been extensively shown that simulation-based training improves self-efficacy and interpersonal relationships, communication skills, collaboration with team members, and the capacity to handle challenging situations [16, 17]. Additional advantages of SBE include practicing invasive and hands-on operations, allowing mistakes to play out naturally, minimizing risks to patients and learners, exposure to rare clinical situations, immediate feedback, and using real equipment [16]. Poor student satisfaction with simulation-based education can lead to a decline in clinical skill proficiency and development [14, 18].

Global studies have shown that developed countries have higher student satisfaction with SBE, with rates exceeding 80% [19, 20]. In contrast, developing nations in Africa, including Ethiopia, there is lower student satisfaction compared to other regions of the continent and overseas [21, 22]. As stated by the WHO, skilled midwives who have undergone the necessary training following international standards can provide 90% of essential care and prevent more than 80% of maternal, neonatal, and stillbirth deaths [23]. Despite the fact that the number of midwives in Ethiopia has more than doubled since 2018, the competency of midwifery students has remained relatively poor [24]; (19–34%) in Ethiopia [25–27]. This luck of needed expertise contributes to client dissatisfaction and jeopardizes patient safety [26, 28].

Currently, both demands from women for high-quality care and the desire from midwives for better education are increasing. As a result, efforts have been made to enhance the quality of midwifery education worldwide. Likewise, the current Ethiopian modularly harmonized curriculum for BSc midwifery education now encompasses collaborative teaching methods such as SBE and PBL (problem-based learning), which promote student cooperation and teamwork throughout the entire program [7].

Although few studies have been conducted in Ethiopia to evaluate midwifery students’ satisfaction with SBE, those studies [5, 18, 22, 28] have predominantly focused on practice-related factors such as the frequency of SBE programs per semester and perceived assistance during practice. Predictors affecting their satisfaction related to the simulation-based education design characteristics as well as the learning environment of SBE have not been fully studied or addressed. Therefore, the aim of this study was to evaluate the magnitude of satisfaction with SBE and the associated factors among undergraduate midwifery students in Amhara region universities in 2022.

## Methods

### Study setting and population

An institution-based cross-sectional study was conducted from August 1–30, 2022 among undergraduate midwifery students at governmental universities in the Amhara region. There are ten governmental universities in the region; of these, eight offer a BSc program in midwifery, and seven of these have 3rd and 4th year students (Bahirdar, Gondar, Debre Tabor, Debre Markos, Debre Birhan, Wollo, and Woldia). More than 800 undergraduate midwifery students were enrolled in the regular program in the region. Third- and fourth-year undergraduate midwifery students in the regular program (*n* = 413) were included in this study. The sample size was proportionally allocated among each university (See supplementary file for the sampling procedure).

### Sampling and data collection procedure

The sample size was determined by using a proportion of satisfaction in simulation-based learning among midwifery students taken from a study conducted in Gondar which was 54.2% [22]. The sample size was determined with a single population proportion formula;

$$\:n\hspace{0.17em}=\hspace{0.17em}\frac{(\frac{\alpha}{2}{z)^{2}\times pq}}{d^{2}}$$

Where,

n- number of sample size.

p- Proportion of satisfaction with simulation-based learning among midwifery students taken from a study conducted in Gondar, 54.2%.

q- Proportion of those who are not satisfied with simulation-based learning (1-p).

d- Permitted marginal error (5%).

Z- Standard normal distribution with a confidence interval of 95%, z = 1.96

$$\:n=\frac{1.962^{2}\times0.542\times(1-0.542)}{0.05^{2}}$$

$$\:n =382$$

After adding a non-response rate of 10%=38; the final total sample size was determined to be 420 among the governmental universities in the Amhara region; eight of them provide midwifery in the BSc program. A simple random sampling technique was used to select study participants after the sample size was proportionally allocated among each university.

Proportional allocation,

$$\:nj=\frac{n\times Nj}{N}$$

Where: nj = is the sample size of the jth university.

Nj = is the population size of the jth university.

n = n1 + n2 + n3 + n4… is the total sample size (420).

N = N1 + N2 + N3 + N4… is the total population size (525).

Data were collected using a structured self-administered questionnaire adapted from the National League for Nursing [29–31]. The tool, prepared in English, and has five parts: sociodemographic information, student satisfaction with SBE, SBE practice, design, and environment. Participants were asked to indicate their level of agreement with listed items on a 5-point Likert scale (1: strongly disagree, 2: disagree, 3: neutral, 4: agree, 5: strongly agree) to measure their satisfaction. To assess the internal consistency of the 5 items of the satisfaction measurement tool, a reliability analysis was conducted, yielding a Cronbach’s alpha value of 0.909. A pretest was performed on 5% of the study subjects at Selalie University, and the tool was subsequently modified for the final data collection. Seven data collectors (BSc midwifery) and two supervisors (MSc in Midwifery) were recruited and training was provided on informed consent, confidentiality, participants’ rights, and the objective of the study. None of the data collectors or supervisors was affiliated with the institutions where the data were collected.

### Measurements and operational definitions

Participants were asked to select their level of agreement with listed items on a 5-point Likert scale (1: strongly disagree, 2: disagree, 3: neutral, 4: agree, 5: strongly agree) to measure the outcome variable satisfaction. Students who scored the mean and above for the 5-item Likert scale listed in the Satisfaction of SBE questionnaires were categorized as satisfied, while those who scored less than the mean for 5-items Likert scale listed in the Satisfaction of SBE questionnaires were categorized as unsatisfied [5, 22].

Independent variables such as simulation-based learning practices include questions that measure students’ active learning, time allotment, frequency of SBE, group size, and collaboration during simulation-based education. Students who scored the mean and above for the 7 items listed under the active learning assessment questions were categorized as those with good active learning practices [31]. Similarly, for determining design-related factors, there were domains such as prebriefing, support, debriefing, feedback, and fidelity. Hence, students who scored the mean and above for 5 items listed under the prebriefing domain assessment were considered those with adequate prebriefing. Likewise support during SBE was measured with 4 items listed under support domain assessment and students who scored the mean and above for those questions were taken as students with adequate support. For measuring debriefing there were 2 items listed under debriefing assessment questions and students who answered ‘Yes’ for both were classified as adequate debriefing. Feedback was also measured with 4 items listed under feedback domain assessment and students who scored mean and above for the questions were grouped as having adequate feedback. Fidelity was measured with 2 items listed under fidelity assessment and students who answered ‘Yes’ for both were themed as those with good fidelity in simulation [30]. Learning environment-related factors were measured with questions concerning the availability of skillful assistants and materials, organization of the simulation room, functionality of equipment, provision of checklists, and availability of instructors.

### Data processing and analysis

Before beginning the analysis procedure, the acquired data were reviewed for completeness. A template format was prepared, and the coded data were entered into Epi-Data version 4.6.0.4 and then exported to SPSS version 26 for analysis (http://www.epidata.dk/download.php*).* Descriptive analysis was employed to describe the percentages and distributions of students’ sociodemographic characteristics and factors associated with their satisfaction with SBE. Binary logistic regression was used to examine the association of independent variables with the dependent variable. Bivariable analysis was conducted to select candidate variables for multivariable analysis at a P-value less than 0.2. Model fitness test was assessed using the Hosmer-Lemeshow goodness-of-fit statistic. Multicollinearity was checked with the variance inflation factor (VIF) test. Crude and adjusted odds ratios (AOR) with corresponding 95% confidence intervals were computed, and a P-value < 0.05 was considered to indicate statistical significance. Finally, the results were presented using tables, figures, and text.

## Results

### Demographic characteristics of students

A total of 413 midwifery students participated in this study, resulting in a response rate of 98.33%. Among the respondents, 195 (47.2%) were male. For most of the participants, the ages of 345 (83.5%) were in the range of 20–24 years with a mean age of 22.94 years (SD ± 2.003). In addition, 168 (40.7%) were in their third year, while 245 (59.3%) were in their fourth year (Table 1).

**Table 1:** Socio-demographic characteristics of undergraduate midwifery students in Amhara region universities, Ethiopia 2022 (*n* = 413)

Variables	Category	Frequency	Percent
Sex	Male	195	47.2%
	Female	218	52.8%
Year of Study	3rd Year	168	40.7%
	4th Year	245	59.3%
Age class	20–24	345	83.5%
	25–29	61	14. 8%
	≥ 30	7	1.7%
University	Gondar	64	15.5%
	Bahir Dar	62	15.0%
	Debretabor	39	9.4%
	Debrebirhan	84	20.3%
	Wollo	58	14.0%
	Debremarkos	51	12.3%
	Woldia	55	13.3%

* Catholic, Jehovah witness

### Midwifery students’ magnitude of satisfaction in sbe

Among the 413 undergraduate midwifery students in Amhara region universities, 350 (84.7%) were satisfied (scoring mean and above) with their simulation-based education. To check for the internal consistency of the 5 items of the satisfaction measurement tool, reliability analysis was conducted, yielding Cronbach’s alpha value of 0.909.

For the five-item Likert scale, 68 (16.5%) participants strongly agreed that the teaching methods used in the simulation were effective. One hundred (24.2%) of them also strongly agreed that the simulated teaching materials were motivating and beneficial to their learning. Moreover, the overall mean SBE score was 3.63 ± 0.991 (Table 2).

**Table 2:** Likert scale information of satisfaction of SBE among midwifery students in Amhara region universities, Ethiopia 2022 (*n* = 413)

Satisfaction of SBE Likert Scale Description		Frequency	Percent
The teaching methods used in simulation were effective.	Strongly disagree	29	7%
	Disagree	50	12.1%
	Neutral	39	9.4%
	Agree	227	55%
	Strongly agree	68	16.5%
The simulation provided me with a variety of learning materials and activities to promote my learning.	Strongly disagree	46	11.1%
	Disagree	67	16.2%
	Neutral	39	9.4%
	*Agree	187	45.3%
	Strongly agree	74	17.9%
I enjoyed how my instructor taught the simulation.	Strongly disagree	26	6.3%
	Disagree	49	11.9%
	Neutral	41	9.9%
	Agree	203	49.2%
	Strongly agree	94	22.8%
The simulated teaching materials were motivating and beneficial to my learning.	Strongly disagree	28	6.8%
	Disagree	51	12.3%
	Neutral	28	6.8%
	Agree	206	49.9%
	Strongly agree	100	24.2%
The way my instructor(s) taught simulation was suitable to the way I learn.	Strongly disagree	26	6.3%
	Disagree	45	10.9%
	Neutral	40	9.7%
	Agree	210	50.8%
	Strongly agree	92	22.3%
	Overall mean=	**3.63 ± 0.991**	

### Simulation-based education practice

The majority 352 (85.2%) of study participants agreed that using simulation activities made their learning time more productive. Two hundred seventy-one students (65.6%) demonstrated overall good active learning practice in SBE. More than half of them, 222 (53.8%), reported that the time allotted to practice a certain procedure was adequate. Two hundred ninety-nine (72.4%) participants exhibited good collaboration practice in SBE (Table 3).

**Table 3:** Simulation based education practice among undergraduate midwifery students in Amhara region universities, Ethiopia 2022 (*n* = 413)

Variables		Frequency	Percent
**Active learning**	**Good**	271	65.6%
	**Poor**	142	34.4%
There were learning objectives for simulation learning sessions.	Yes	406	98.3%
	No	7	1.7%
I actively participated before, during, or after the simulation.	Yes	390	94.4%
	No	23	5.6%
I had the chance to discuss concepts taught in simulation with my instructor.	Yes	355	86%
	No	58	14%
I learned from the comments made by the teacher before, during, or after the simulation.	Yes	362	87.7%
	No	51	12.3%
Using simulation activities made my learning time more productive.	Yes	352	85.2%
	No	61	14.8%
There were enough opportunities in the simulation to understand the materials.	Yes	298	72.2%
	No	115	27.8%
I had adequate opportunity to practice in simulation learning sessions.	Yes	271	65.6%
	No	142	34.4%
I had adequate time to practice a certain procedure in simulation.	Yes	222	53.8%
	No	191	46.2%
During simulation, the group size was appropriate for my learning.	Yes	261	63.2%
	No	152	36.8%
**Collaboration**	**Good**	299	72.4%
	**Poor**	114	27.6%
I had the chance to work with my peers.	Yes	356	86.2%
	No	57	13.8%
I had the opportunity during simulation to discuss concepts taught in the course with both the teacher and peers.	Yes	318	77%
	No	95	23%
Do you think there were adequate simulation sessions in your education?	Yes	228	55.2%
	No	185	44.8%
Number of simulation sessions per semester	< 15	222	53.8%
	≥ 15	191	46.2%

### Simulation-based education design

With respect to SBE designs, 291 (70.5%) participants reported that there was adequate prebriefing. Only 250 (60.5%) of midwifery students felt that adequate support was given in simulation education. In addition, 278(67.3%) reported that there was adequate debriefing of SBE. Two hundred twenty-nine (55.4%) of the students reported that the feedback given after simulation-based learning was adequate. The students also indicated that there was good fidelity of simulation-based education 291 (70.5%) (Table 3).

### SBE environment-related characteristics

The majority of the respondents, 384(93%), reported that instructors were available during their simulation learning. In addition, 276(66.8%) stated that lab assistants were skillful. Two hundred seventy-nine (67.6%) students agreed that their learning environment had the necessary materials for conducting simulation learning. Three hundred thirty-five (81.1%) of them also noted the presence and provision of checklists in the simulation room. Two hundred ninety-nine (72.4%) students reported that the simulation room and materials were well organized (Table 3).

### Factors associated with students’ satisfaction with SBE

After controlling for the effects of other covariates, fifteen variables (year of study, active learning, collaboration, adequacy of sessions, prebriefing, support, debriefing, feedback, fidelity, instructor availability, skillful lab assistants, availability of materials, provision of checklists, functionality of materials, and organization of rooms) that were significantly associated with satisfaction with SBE according to the bivariable analysis (with P-value less than 0.2) were fitted in the multivariable logistic regression model for further investigation. Among these predictor variables year of study, support, instructor’s availability, and provision of checklists were significantly associated with students’ satisfaction with SBE.

This study revealed that the odds of being satisfied with SBE among 4th-year midwifery students were 2.93 times higher than 3rd-year students [AOR: 2.93; 95% CI (1.53–5.63)]. The result also showed that the odds of being satisfied with SBE among students with adequate support were 2.90 times higher than students receiving inadequate support [AOR: 2.90; 95% CI (1.21–6.92)]. The odds of being satisfied with SBE among students with available instructors in a simulation learning environment were 2.86 times higher than those students with no instructors during simulation [AOR = 2.86, 95%t CI (1.07–7.59)]. In addition, the odds of being satisfied with SBE among students in a simulation learning environment where checklists were provided were 2.32 times higher than those students in a simulation learning environment where checklists were not provided [AOR: 2.32; 95% CI (1.14–4.73)] (Table 4, Table 5 and Table 6).

**Table 4:** Simulation based education design characteristics among undergraduate midwifery students in Amhara region universities, Ethiopia 2022 (*n* = 413)

Variables		Frequency	Percent
**Prebriefing**	**Adequate**	291	70.5%
	**Inadequate**	122	29.5%
The teacher explained learning objectives for simulation at the beginning.	Yes	382	92.5%
	No	31	7.5%
The objectives were clear and easy to understand.	Yes	368	89.1%
	No	45	10.9%
During simulation, checklists were elaborated and explained.	Yes	366	88.6%
	No	47	11.4%
The cues were appropriate and able to promote my understanding.	Yes	373	90.3%
	No	40	9.7%
I clearly understood the purpose and objectives of the simulation.	Yes	367	88.9%
	No	46	11.1%
**Support**	**Adequate**	**250**	**60.5%**
	**Inadequate**	**163**	**39.5%**
During simulation, I was able to get necessary help in use of equipment.	Yes	317	76.8%
	No	96	23.2%
Support was offered in a timely manner.	Yes	304	73.6%
	No	109	26.4%
I felt supported by teacher’s assistance during simulation.	Yes	331	80.1%
	No	82	19.9%
Instructors at simulation guided and showed necessary skill.	Yes	337	81.6%
	No	76	18.4%
**Debriefing**	**Adequate**	**278**	**67.3%**
	**Inadequate**	**135**	**32.7%**
After simulation, I was allowed to discuss my concerns with the teacher and peers.	Yes	326	78.9%
	No	87	21.1%
I had an opportunity to put more thought into my comments during the debriefing	Yes	317	76.8%
	No	96	23.2%
**Feedback**	**Adequate**	**229**	**55.4%**
	**Inadequate**	**184**	**44.6%**
There was an opportunity after simulation to obtain feedback from the teacher.	Yes	290	70.2%
	No	123	29.8%
There was an opportunity after simulation to obtain feedback from other students.	Yes	331	80.1%
	No	82	19.9%
Feedback provided was constructive.	Yes	344	83.3%
	No	69	16.7%
Feedback was provided in a timely manner.	Yes	307	74.3%
	No	106	25.7%
**Fidelity**	**Good**	**291**	**70.5%**
	**Poor**	**122**	**29.5%**
Real life situations and variables are built into the simulation scenario.	Yes	326	78.9%
	No	87	21.1%
The materials in simulation resembled real-life situations.	Yes	319	77.2%
	No	94	22.8%

**Table 5:** Simulation based learning environment characteristics among undergraduate midwifery students in Amhara Region universities, Ethiopia 2022 (*n* = 413)

Variables		Frequency	Percent (%)
Instructors were available during simulation.	Yes	384	93%
	No	29	7%
Lab assistants were skillful.	Yes	276	66.8%
	No	137	33.2%
Necessary materials were present in the simulation room.	Yes	279	67.6%
	No	134	32.4%
Checklists were provided.	Yes	335	81.1%
	No	78	18.9%
Materials in simulation room were functional.	Yes	300	72.6%
	No	113	27.4%
There was good organization of simulation room and materials.	Yes	299	72.4%
	No	114	27.6%

**Table 6:** Bivariate and multivariate analysis of factors affecting satisfaction of SBE among undergraduate midwifery students in Amhara region universities, Ethiopia 2022

Variables		Satisfaction of SBE				
		**Satisfied**	**Unsatisfied**	**COR (CI = 95%)**	**AOR (CI = 95%)**	**PV**
Year of study	**Fourth Year**	221(90.2%)	24(9.8%)	2.78(1.60–4.83)***	2.93 (1.53–5.63)**	**0.001**
	Third Year	129(76.8%)	39(23.2%)	1	1	
Active Learning	Good	241 (88.9%)	30 (12.1%)	2.43(1.41–4.18)**	0.559 (0.23–1.33)	0.191
	Poor	109(76.8%)	33(23.2%)	1	1	
Collaboration	Good	267(89.3%)	32(9.7%)	3.11(1.79–5.41)***	1.82 (0.86–3.85)	0.117
	Poor	83(72.8%)	31(27.2%)	1	1	
Pre-briefing	Adequate	264(90.7%)	27(9.3%)	4.09(2.34–7.13)***	1.84 (0.91–3.68)	0.085
	Inadequate	86(70.5%)	36(29.5%)	1	1	
Support	**Adequate**	232(72.4%)	18(27.6%)	4.91(2.72–8.86)***	2.90 (1.21–6.92)*	**0.016**
	Inadequate	118(92.8%)	45(7.2%)	1	1	
De-briefing	Adequate	245(88.1%)	33(11.9%)	2.12(1.23–3.65)*	0.90 (0.42–1.93)	0.803
	Inadequate	105(77.8%)	30(22.2%)	1	1	
Feedback	Adequate	204(89.1%)	25(10.9%)	2.12(1.22–3.67)*	0.70 (0.32–1.53)	0.377
	Inadequate	146(79.3%)	38(20.7%)	1	1	
Fidelity	Good	259(89%)	32(11%)	2.75(1.59–4.77)***	1.43 (0.67–3.05)	0.349
	Poor	91(74.6%)	31(25.4%)	1	1	
Instructors Availability	**Yes**	331(86.2%)	53(13.8%)	3.28(1.44–7.45)*	2.86 (1.07–7.59)*	**0.035**
	No	19(65.5%)	10(34.5%)	1	1	
Skillful Lab assistants	Yes	240(87%)	36(13%)	1.63(0.94–2.83)	1.28 (0.65–2.54)	0.466
	No	110(80.3%)	27(19.7%)	1	1	
Availability of Materials	Yes	242(86.7%)	37(13.3%)	1.57(0.90–2.73)	0.63 (0.31–1.28)	0.205
	No	108(80.6%)	26(19.4%)	1	1	
Provision of Checklists	**Yes**	298(89%)	37(11%)	4.02(2.25–7.20)***	2.32 (1.14–4.73)*	**0.020**
	No	52(66.7%)	26(33.3%)	1	1	
Functionality of Materials	Yes	265(88.3%)	35(11.7%)	2.49(1.43–4.33)**	1.13 (0.53–2.40)	0.744
	No	85(75.2%)	28(24.8%)	1	1	
Organization of Room	Good	267(89.3%)	32(10.7%)	3.11(1.79–5.41)***	1.56 (0.72–3.38)	0.250
	Poor	83(72.8%)	31(27.2%)	1	1	

*P* < 0.05*, *P* < 0.001**, *P* < 0.0001***, CI = Confidence Interval, COR = Crude Odd Ratio, AOD = Adjusted Odd Ratio

## Discussion

The result of this study revealed 84.7% satisfaction with simulation-based education (95% CI; 81.1-88.3%). This finding is higher than findings from a study conducted at Gondar University (54.2%) and a study done at Harar Health Science College and Dire Dawa University (70.95%) satisfaction with SBE [5, 22]. These findings are also higher compared to a study conducted in Egypt, which showed 68% satisfaction [21]. The discrepancy might be due to the difference in study setting facilities and sample sizes. In addition this discrepancy could be attributed to modifications in the curriculum implemented (modularly harmonized curriculum for Midwifery education). As a key aspect of their efficient usage, simulation-based exercises are highly integrated into the modularly harmonized midwifery education curriculum [7, 32, 33]. The inconsistency might be due to the differences in the study setting and the use of a specific simulator type in the study conducted in Egypt.

The result of this study is consistent with findings of studies performed in developed countries, which reported that85% of students in Saudi Arabia [20] and 87% in Gardner-Webb University in United States were satisfied [34]. This similarity might be due to technological advancements in diverse simulation technologies.

Furthermore, year of study, support, availability of instructors, and provision of checklists were found to be significantly associated with satisfaction with simulation-based education. In this study, the odds of being satisfied with SBE among midwifery students who were attending their 4th year were 2.93 times higher than those among 3rd year students, which represent a new finding of this study.

This result is inconsistent with findings from a study conducted at Gondar University [22]. This result is also inconsistent with a study done in Saudi Arabia, which showed that there was no significant association between student satisfaction and year of study [20]. The possible reason might be that 3rd year students may face anxiety due to limited prior experience and exposure to simulation practices leading to lower satisfaction with simulation learning activities.

According to the findings of this study, the odds of being satisfied with SBE among students who received adequate support were 2.90 times higher than among those who received inadequate support during conducting simulations. This result is consistent with the findings of studies conducted in Harar, Dire Dawa, and Gondar [5, 22]. Similarly, this finding aligns with a study conducted in Canada [35]. A possible explanation for this might be that students who get adequate support will have the awareness and assistance in skills to meet the desired objectives in simulation-based education. A supportive learning environment in simulation provides an opportunity to ensure that training addresses affective issues, as it deliberately places the student’s needs at the center of attention [32, 36].

This study revealed that the odds of being satisfied with SBE among students with available instructors in a simulation learning environment were 2.86 times higher than among those with no instructors during simulation. This finding is supported by qualitative studies in Ethiopia [18, 28]. The possible reason might be that the students will experience and benefits from both theoretical and practical knowledge in the instructor’s presence. This might be further explained by actions taken during the simulation experience, the facilitator adjusts teaching tactics and guides the scenario in response to growing participant demands which are important to the achievement of learning objectives [32, 37].

In addition, this study also showed that midwifery students in a simulation learning environment where checklists were provided were 2.32 times more likely to be satisfied than students in an environment where checklists were not provided, which is also a new finding of this study. This might be explained by a guided learning process through having a clear step-by-step procedure outline at hand will enhance their satisfaction with simulation education as well as the assessment of practical skills [36, 38].

### Limitation

This study was conducted at the regional level using a large sample size and probability sampling method to minimize selection bias and to ensure its generalizability to midwifery students studying at universities found in different regions of Ethiopia. To minimize information bias, a pretested, valid, and reliable questionnaire was used to collect data. Study participants were also limited in the use of exclusion criteria to those who were in clinical practice during the data collection period to reduce recall bias. Similarly, the objective of the study was clearly described to the study participants, and the confidentiality of their responses was assured to encourage them to provide their genuine response. In addition, data quality was maintained throughout, and an appropriate statistical analysis method was used with a strong model goodness of fit test. However, further research using qualitative approaches should be used.

## Conclusion

Generally, the results of this study revealed that the satisfaction of undergraduate midwifery students with simulation-based education in Amhara region Governmental Universities was higher compared to previous studies conducted in Ethiopia. Predictor variables such as year of study, support, instructor’s availability, and provision of checklists were significantly associated with students’ satisfaction with SBE. Considering the findings of this study, it is recommended that midwifery departments achieve better results by increasing the support given by staff in simulation-based education to enhance students’ satisfaction, ensuring instructors’ availability during simulation-based education sessions, and improving the utilization and provision of checklists to students as much as possible. In addition, for future researchers, it is better to investigate the quality of simulation-based education in midwifery courses provided by Ethiopian universities.

## Electronic supplementary material

Below is the link to the electronic supplementary material.

Supplementary Material 1

## Data Availability

The data sets collected and analyzed for the current study are available from the corresponding author and can be obtained at a reasonable request.

## Abbreviations

AOR: Adjusted odds ratio
CI: Confidence interval
ICM: International confederation of Midwives
SBE: Simulation-based education
SBL: Simulation-based learning
SD: Standard deviation
SDG: Sustainable development goals
SPSS: Statistical package for social sciences
