# Supplementary material for: Midwifery students’ satisfaction with simulation-based education and associated factors among governmental universities in Amhara region, Ethiopia 2022
Source: BMC Med Educ. 2024 Sep 30;24:1070. doi: 10.1186/s12909-024-05974-2 (PMC11443949; doi:10.1186/s12909-024-05974-2)
Supplement: Supplementary file 1 — Supplementary Material 1 [file 12909_2024_5974_MOESM1_ESM.docx]

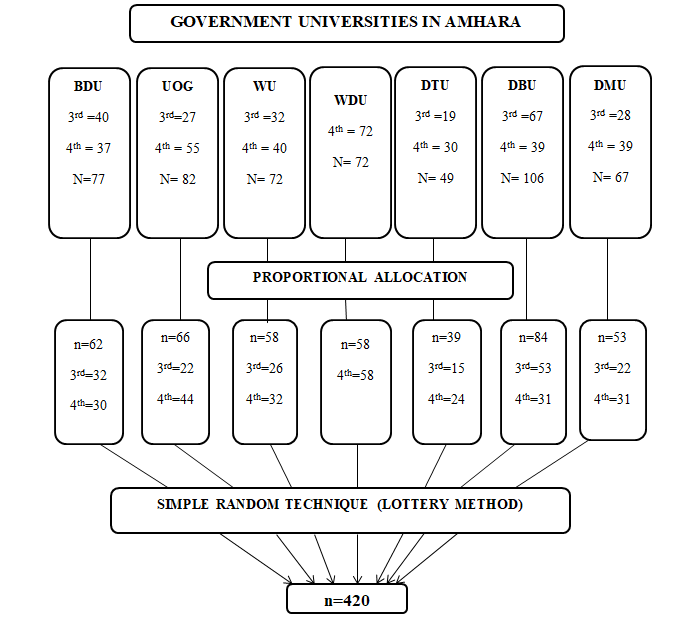


- S Figure 1 - ''Schematic diagram of sampling procedure for Students' satisfaction of SBE among undergraduate Midwifery Students in Amhara region Universities, Ethiopia 2022''
